# Endoresection in Choroidal Melanoma: Outcomes of Intentional Incomplete Tumor Removal

**DOI:** 10.3390/curroncol32120688

**Published:** 2025-12-04

**Authors:** Alexander Anduaga-Beramendi, Marta Caminal-Caramés, Daniel Lorenzo, Estefanía Cobos, Milagros Mateos-Olivares, Pere Garcia-Bru, Rahul Morwani, Juan Santamaría, Olga Garcia-Garcia, Luis Arias, Josep M. Caminal

**Affiliations:** 1 Ophthalmology Department, Bellvitge University Hospital, 08907 L’Hospitalet de Llobregat, Spain; 2 Facultat de Medicina i Ciències de la Salut, Universitat de Barcelona (UB), c. Casanova, 143, 08036 Barcelona, Spain; 3 Ophthalmology Department, Santa Creu and Sant Pau Hospital, 08041 Barcelona, Spain

**Keywords:** uveal melanoma, endoresection, adjuvant radiotherapy, survival, ocular oncology

## Abstract

To assess the outcomes of a modified surgical approach for the treatment of uveal melanoma involving endoresection with intentional residual tumor at the margins, combined with adjuvant ruthenium-106 brachytherapy. This technique aims to reduce surgical morbidity, while preserving visual function and maintaining effective local tumor control and survival. We conducted a retrospective observational study including 33 patients with choroidal melanoma treated between January 2017 and August 2024 at a single tertiary ocular oncology center in Spain. Patients underwent pars plana vitrectomy and endoresection leaving residual tumor followed by ruthenium-106 brachytherapy. Clinical, functional, and oncological outcomes were analyzed, including tumor recurrence, metastasis, visual acuity, complications, and cytogenetic findings. Kaplan–Meier analysis was used to estimate survival and recurrence rates. After a mean follow-up of 41.7 months, local tumor recurrence occurred in 2 patients (6.06%) and enucleation was performed in 1 patient (3.03%). Two patients (6.06%) developed metastases, with one disease-specific death, resulting in a 5-year survival rate of 97%. Visual acuity of 20/200 or better was preserved in 60.61% of patients. The most frequent complications were retinal detachment (36.36%) and macular edema (45.45%). Cytogenetic analysis showed a significant association between chromosome 1p loss and both recurrence and metastasis (*p* = 0.032). No cases of phthisis bulbi or severe hypotony were observed. This modified endoresection technique with intentional tumor residuals and adjuvant ruthenium-106 brachytherapy offers a safe and function-preserving option for selected patients with choroidal melanoma. It achieves good tumor control and visual outcomes, with a low rate of enucleation and metastasis. Further studies are required to validate its long-term efficacy.

## Simple Summary

Choroidal melanoma is a malignant eye tumor in which preserving vision and controlling the disease are challenging. This study describes a modified endoresection approach that intentionally leaves small amounts of tumor tissue, especially at the posterior border, to reduce eye damage, combined with brachytherapy to eliminate residual cancer cells. By avoiding complete tumor removal, the technique aims to reduce surgical complications and improve visual outcomes without compromising safety. The findings suggest that this surgical technique could offer a promising alternative for patients whose priority is preserving vision, with low recurrence and metastasis rates.

## 1. Introduction

Uveal melanoma is the most frequent primary intraocular malignant tumor in adults, with an incidence between 1.3–8.6 cases per million per year in Europe [1,2]. The most common cause of death in these patients is distant metastasis, which occurs in 50% of patients with large uveal melanomas, thus making local tumor control critical [3,4,5]. The optimal treatment is controversial, so the approach must be individualized according to tumor and patient related factors [6]. Over the past few decades, the management of uveal melanoma has shifted significantly, moving from enucleation to eye-preserving treatment modalities. Although these techniques demonstrate high rates of local tumor control, they remain associated with complications that can lead to reduced visual acuity [7].

Radiation therapy is the most widely used treatment in most centers, especially for small- and medium-sized tumors [8]. However, it is associated with complications, such as radiation retinopathy, optic neuropathy, cataract, toxic tumor syndrome and secondary glaucoma [9,10]. Selected patients can benefit from other treatments such as surgical resection, especially in large uveal melanomas with pronounced height and a small base diameter [6,11,12]. Transscleral resection or ab externo technique for iris, ciliary body or anterior choroidal tumors and endoresection or ab interno technique for posterior choroidal tumors [6,13]. These techniques enable tumor control while preserving the globe, maximizing visual acuity, and reducing the complications associated with radiotherapy [2,14]. They also provide tumor tissue for histopathologic and cytogenetic analysis.

Endoresection is a complex procedure, and it is only performed in specialized centers, as it carries early and late postoperative risks [13,15]. During the procedure, a vitreous cutter is used to remove the tumor from the apex until the scleral bed is clear [6]. Wide removal of the tumor margins during surgery increases the risk of hemorrhage, despite the use of preventive measures such as increased intraocular pressure and photocoagulation. Scleral bed hemorrhage involving the fovea remains one of the most frequent causes of low visual acuity [6]. It is unknown how much residual tumor can be left for the procedure to remain successful but leaving residual tumor at the margins of the tumor, especially near the fovea, may make the procedure less aggressive and potentially allow better preservation of visual acuity. Biewald and colleagues reported cases of endoresection where residual tumor was left, not exceeding 3 mm in height, along with adjuvant radiotherapy, achieving encouraging results [16]. Nowadays, adjuvant radiotherapy is often associated with endoresection to treat any tumor remnants, which are assumed to be minimal [2,6,10,16,17,18].

The aim of this study is to describe a technique for the treatment of uveal melanoma by modified endoresection leaving residual tumor at the margins, combined with adjuvant brachytherapy using ruthenium-106 (^106^Ru) plaque, and to evaluate its results in terms of tumor control, functional outcome, local complications and survival.

## 2. Materials and Methods

### 2.1. Study Design

We conducted a single center observational retrospective study on patients diagnosed with choroidal melanoma who underwent endoresection with residual margins from January 2017 through August 2024 at the Ocular Oncology Unit of the Ophthalmology Department of Bellvitge University Hospital. Conventional endoresection was not performed during this period. Patient confidentiality was protected by national data confidentiality laws. This study was approved by the Clinical Research Ethics Committee of Bellvitge University Hospital. Written informed consent was obtained from all patients, who were fully informed of the procedure, the controversy around the surgical procedure and other potential treatment options. Patients included in the study had choroidal melanoma and were treated with endoresection with tumor preservation near critical visual structures combined with adjuvant ^106^Ru brachytherapy. Critical visual structures were defined as the optic nerve and the fovea. Exclusion criteria included distant metastasis, scleral involvement at diagnosis, ciliary body involvement, previous treatment for uveal melanoma and follow-up of less than 6 months.

### 2.2. Data Collection, Treatment and Follow-Up

A complete preoperative ophthalmic examination was performed on both eyes, including best-corrected visual acuity (BCVA), retinography, and A- and B-scan ultrasonography to assess maximum tumor height, basal diameter, and distances to the fovea and optic nerve. Metastatic disease was ruled out by a medical oncologist through serum biochemistry, liver ultrasound, and chest radiography.

**Figure 1:**
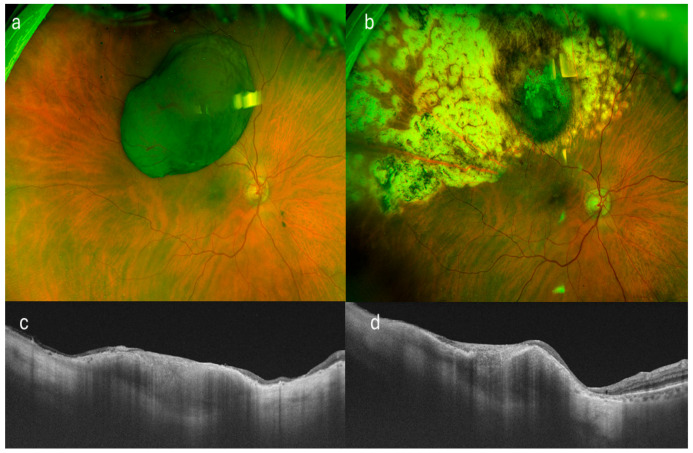
Multimodal imaging of a patient with superotemporal uveal melanoma treated by endoresection with residual tumor margins. (**a**) Color fundus photograph prior to surgical intervention, showing the pigmented choroidal lesion. (**b**) Postoperative fundus image following endoresection, revealing the surgical bed with visible residual tumor at the margins. (**c**,**d**) Swept-source optical coherence tomography (SS-OCT) scans in transverse and longitudinal planes, respectively, highlighting the residual tumor tissue at the scleral bed. Note the small remaining tumor in both sections.

**Figure 2:**
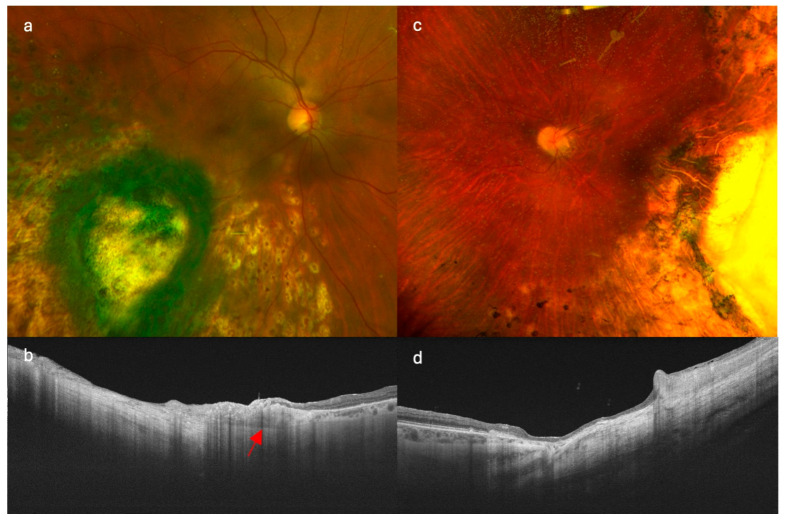
Comparison of imaging findings in patients treated with different endoresection techniques. (**a**,**b**) show retinography and swept-source optical coherence tomography (SS-OCT), respectively, in a patient treated with modified endoresection preserving residual tumor margins with adjuvant brachytherapy. (**c**,**d**) correspond to a patient treated with conventional complete endoresection with adjuvant brachytherapy. Note the preserved choroidal tissue at the tumor margins in image (**b**) (red arrow), particularly in the posterior region, which contrasts with the complete resection observed in (**d**).

In phakic patients, the procedure began with lens phacoemulsification followed by intraocular lens implantation. A 23-gauge pars plana vitrectomy was then performed using a panoramic viewing system, with induction of posterior vitreous detachment. In cases with an intact retina, a peripheral retinectomy was carried out to allow folding of the retina away from the tumor. If retinal invasion was present, a transretinal tumor resection was performed. Endolaser photocoagulation was applied 2 mm beyond the tumor margins. Intraocular pressure was temporarily elevated to 60–70 mmHg to reduce bleeding, and the tumor was excised piecemeal from apex to base using a vitreous cutter. In all patients, complete tumor resection was intentionally avoided near critical structures, especially at the posterior border (Figure 1 and Figure 2). Tumor remnants up to 3 mm in height near critical visual structures were tolerated and managed with planned adjuvant brachytherapy, prescribed to a depth of 3 mm from the scleral surface. The residual tumor height was estimated under microscopic visualization during the procedure. A 3 mm threshold was selected based on previous reports describing successful management using this criterion [16].

Perfluorocarbon liquid was used to reattach the retina, followed by completion of endolaser photocoagulation around the tumor site. A direct exchange of perfluorocarbon with silicone oil was then performed.

A ^106^Ru plaque was sutured to the sclera at the tumor base, delivering a total dose of 75 Gy to a depth of 3 mm, with a minimum safety margin of 2 mm. Cryotherapy was applied at the sclerotomy sites. Throughout all procedures, patients’ systemic blood pressure remained within normal limits. All surgeries were performed by two senior vitreoretinal surgeons (J.M.C. and D.L.). Tumor samples were obtained intraoperatively for histopathological and cytogenetic analysis. Genetic profiling was performed using the SALSA^®^ MLPA^®^ Probemix P027-B1 Uveal Melanoma kit (MRC-Holland, Amsterdam, The Netherlands) and analyzed with Coffalyser.Net MLPA^®^ analysis software (DAT v8, MRC-Holland). Sequence analysis of selected mutations was carried out using the PyroMark Q24 System (Qiagen, Hilden, Germany).

Postoperative follow-up visits were scheduled at day 1, week 1, and at 1, 3, and 6 months. Thereafter, follow-up evaluations, including systemic screening for metastasis, were conducted every 6 months for the first 5 years and annually thereafter. Data collected included local tumor control, anterior and posterior segment complications, need for enucleation, development of metastases, and survival status. Follow-up was extended until the last entry in each patient’s medical record.

### 2.3. Statistical Analysis

Descriptive statistics were pre-specified. For continuous variables, the mean and standard deviation were calculated when data were normally distributed, and the median with interquartile range otherwise. Normality was assessed with Shapiro–Wilk test and Q-Q plots. Categorical variables were summarized using absolute and relative frequencies. Bivariate analyses were conducted to explore associations between clinical variables and outcomes. For quantitative variables, comparisons were made using Student’s *t*-test (with Welch’s correction when variances were unequal per Levene’s Test) or the Mann–Whitney *U* test, as appropriate. Categorical variables were compared using the chi-square test or Fisher’s exact test, as appropriate. Kaplan–Meier survival curves were generated to describe time-to-event outcomes. All statistical tests were two-sided, and a *p*-value < 0.05 was considered statistically significant. Statistical analyses were performed using Stata v15.1 (StataCorp LLC., College Station, TX, USA).

## 3. Results

### 3.1. Patient and Tumor Characteristics

A total of 33 patients with choroidal melanoma who underwent endoresection with residual margins and adjuvant brachytherapy in the same procedure between January 2017 and August 2024 were evaluated. The mean age was 59.78 years (Standard deviation [SD], 12.58). Of the 33 patients, 20 (60.61%) were male and 13 (39.39%) females. The right eye was affected in 21 cases (63.64%). The mean visual acuity was 0.43 (SD, 0.38) on the Snellen chart and median of 0.5 (IQR, 0.90) on LogMAR. Visual acuity at presentation was 20/200 or better in 25 patients (75.76%), and worse than 20/200 in 8 patients (24.24%). The mean tumor height was 9.05 mm (SD, 1.98), with a mean maximum basal diameter of 12.05 mm (SD, 2.24). The average horizontal and vertical base diameters were 11.56 mm (SD, 2.47) and 11.19 mm (SD, 2.38), respectively. The mean distances from the tumor to the fovea and optic nerve were 5.05 mm (SD, 2.70) and 4.38 mm (SD, 3.60), respectively. According to the Collaborative Ocular Melanoma Study (COMS) classification, 25 tumors (75.76%) were medium-sized and 8 (24.24%) were large. Based on the 8th edition American Joint Committee on Cancer staging system (AJCC), 11 tumors (33.33%) were classified as T2 and 22 (66.67%) as T3. Exudative retinal detachment (RD) was present in 26 patients (78.79%). The most common tumor location was inferotemporal (8 patients, 24.24%), followed by superonasal (6 patients, 18.18%) and temporal (5 patients, 15.15%). Bruch’s membrane rupture was observed in 31 cases (93.94%). The mean follow-up period was 41.70 months (SD, 17.98) (Table 1 and Table 2).

### 3.2. Adjuvant Brachytherapy

Regarding adjuvant brachytherapy, the most used plaque was the 18 mm ^106^Ru model, applied in 19 patients (57.58%), followed by the 13 mm model in 12 patients (36.36%) and the 20 mm model in 2 patients (6.06%). The mean radiation dose delivered was 75.03 ± 2.31 Gy at a depth of 3 mm from the tumor base. Only one patient (3.03%) required a second brachytherapy treatment due to local tumor recurrence.

### 3.3. Sample Analysis

Genetic analysis was performed on tissue samples obtained during surgery. In 2 of the 33 patients, analysis could not be completed due to insufficient tissue. A *GNAQ* mutation was identified in 18 patients (58.06%) and a *GNA11* mutation in 12 patients (38.71%). *SF3B1* gene analysis was performed in 29 samples, revealing mutations in 9 cases. No other genes were analyzed. No mutations were significantly associated with recurrence or metastasis.

Cytogenetic analysis could not be performed in 3 of the 33 patients due to insufficient tissue. Among the remaining 30 patients, chromosome 3 status revealed complete monosomy in 6 patients (20.0%), while 2 patients (6.7%) presented partial loss of the short arm (3p). Alterations in chromosome 6 were the most frequently detected: 2 patients (6.7%) had complete gain of chromosome 6, 13 (43.3%) had 6p gain, and 3 (10.0%) had 6q loss. Notably, two of the three patients with 6q loss also exhibited concurrent 6p gain.

Regarding chromosome 8, 5 patients (16.7%) showed 8q gain, 1 patient (3.3%) had 8q loss, and 1 patient (3.3%) presented trisomy 8. Additionally, 1p loss was detected in 6 patients (20.0%). Loss of chromosome 1p was significantly associated with local tumor recurrence and metastasis (fisher’s exact test, *p* = 0.032). No other chromosomal alterations had a statistically significant association with recurrence or metastasis.

### 3.4. Local Tumor Control and Globe Salvage

Two patients (6.06%) experienced local tumor recurrence at the surgical margins during the follow-up period. A greater distance between the tumor and both the fovea (Mann–Whitney *U* test, *p* = 0.041) and the optic disc (Mann–Whitney *U* test, *p* = 0.047) was significantly associated with local recurrence. Tumor height and largest basal diameter were not associated with either local recurrence or metastasis. No cases showed diffuse dissemination of tumor cells (Table 3).

**Figure 3:**
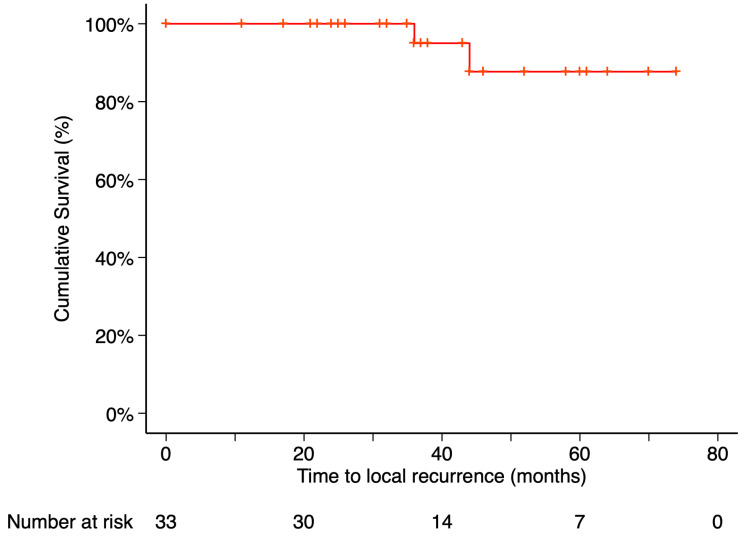
Kaplan–Meier curve of local recurrence–free survival. Local recurrence–free survival was defined as the time from treatment initiation to the first confirmed local recurrence, with patients without recurrence censored at their last follow-up visit. Tick marks indicate censored data. The number of patients at risk at each time point is shown below the plot.

**Figure 4:**
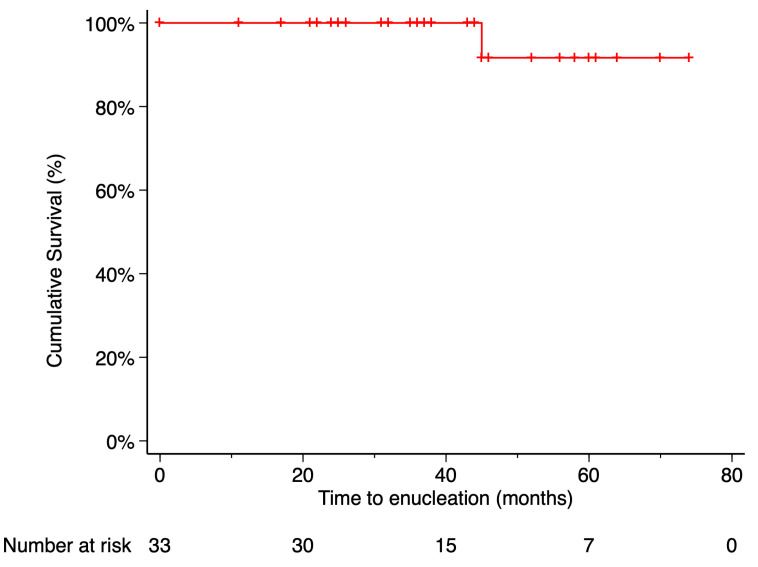
Kaplan–Meier curve of enucleation-free survival. Enucleation-free survival was defined as the time from treatment initiation to enucleation, with patients who did not require enucleation censored at their last follow-up visit. Tick marks indicate censored data. The number of patients at risk at each time point is shown below the plot.

The Kaplan–Meier estimated local recurrence-free survival rate was 87.69% at 5 years post-treatment (Figure 3). Time to local recurrence was 44 months in the first case and 36 months in the second. Prior to local tumor recurrence, the first patient developed a RD with proliferative vitreoretinopathy (PVR). Due to severely reduced vision, enucleation was performed. Histopathological examination of the enucleated eye revealed limited histological extrascleral spread, and the patient subsequently received orbital radiotherapy. This patient has been followed for more than six years without evidence of metastatic disease. Notably, this was the only patient in the study who did not retain the ocular globe at the end of the follow-up period (Figure 4). In the second case, local tumor recurrence was detected during follow-up and managed with a second 20 mm ^106^Ru plaque, delivering a dose of 75 Gy at a depth of 3 mm from the tumor base. Tumor growth was successfully controlled. None of the patients who developed local recurrence presented metastasis or died by the end of the follow-up period.

### 3.5. Metastasis and Death

**Figure 5:**
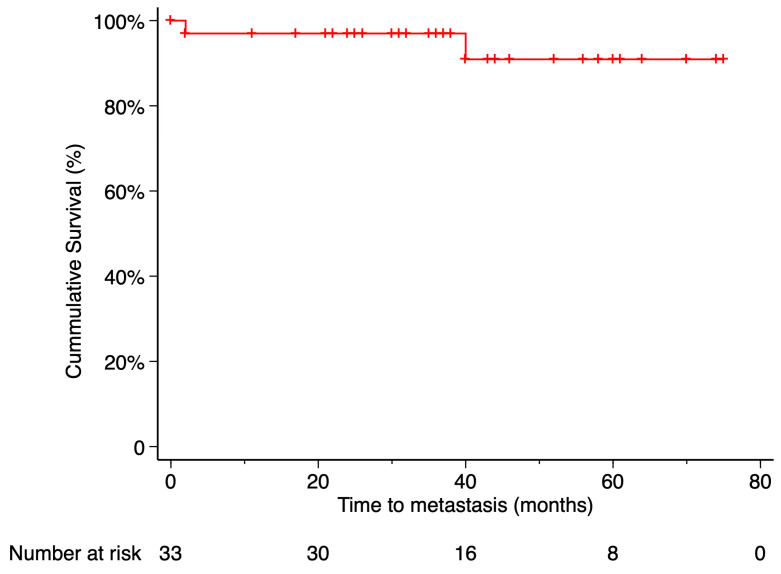
Kaplan–Meier curve of metastasis-free survival. Metastasis-free survival was defined as the time from treatment initiation to the first confirmed metastatic event, with patients who did not develop metastasis censored at their last available follow-up. Tick marks indicate censored data. The number of patients at risk at each time point is shown below the plot.

**Figure 6:**
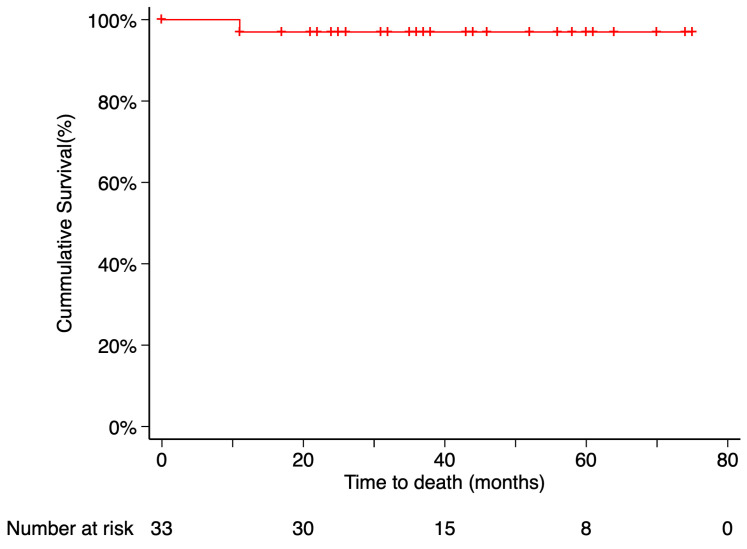
Kaplan–Meier curve of disease-specific survival. Disease-specific survival was defined as the time from treatment initiation to death attributable to the primary tumor, with patients who remained alive censored at their last follow-up visit. Tick marks indicate censored data. The number of patients at risk at each time point is shown below the plot.

Metastasis occurred in 2 patients (6.06%), with the liver being the site of involvement in both cases. The Kaplan–Meier estimated metastasis-free survival rate at 5 years post-treatment was 90.91%. Time from endoresection to metastasis was 2 months in one case and 40 months in the other. Only the death of the first patient was recorded during the follow-up period. The time from metastasis diagnosis to death was 9 months. According to Kaplan–Meier analysis, the estimated 5-year overall survival was 97% (Figure 5 and Figure 6).

The patient who developed metastasis 2 months after endoresection died during follow-up and had a Class D metastasis profile according to The Cancer Genome Atlas (TCGA) classification, based on cytogenetic alterations [19]. The other patient who developed metastasis had a Class C profile according to the same classification.

### 3.6. Visual Acuity

**Figure 7:**
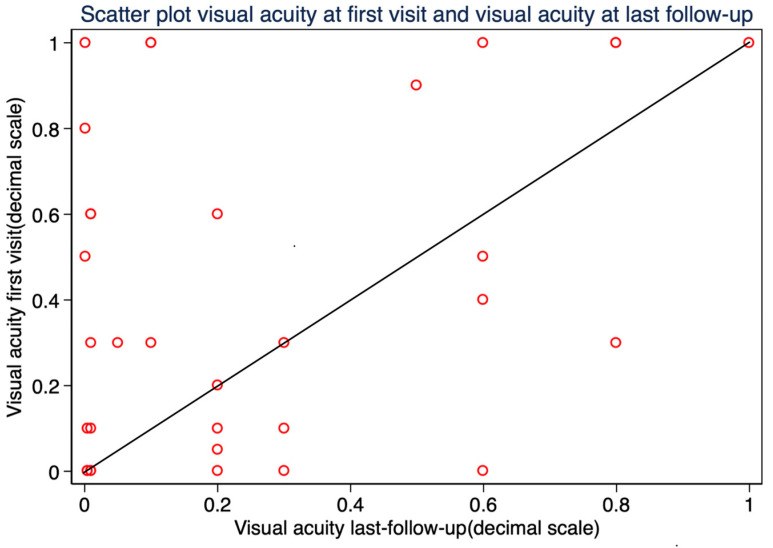
Scatter plot of visual acuity (decimal scale) at baseline versus final follow-up.

The mean best-corrected visual acuity (BCVA) at the time of diagnosis was 0.43 ± 0.39 (SD), with 20/40 or better in 14 patients (42.42%) and 20/200 or better in 25 patients (75.76%). At the last follow-up, the median BCVA was 0.20 ± 0.49 (IQR), with 20/40 or better in 9 patients (27.27%) and 20/200 or better in 20 patients (60.61%). Final BCVA improved in 14 patients (42.42%), remained unchanged in 3 patients (9.09%), and worsened in 16 patients (48.48%) compared to preoperative values. (Figure 7) BCVA at the last follow-up was significantly worse in patients who developed RD after endoresection (Mann–Whitney *U* test, *p* = 0.003). No statistically significant association was found between final visual acuity and other variables: tumor thickness, tumor diameter, tumor distance to the optic nerve, and tumor distance to the fovea.

### 3.7. Treatment Complications

Major revision was defined as any additional intraocular procedure beyond silicone oil removal. It was required in 12 patients (36.3%), with retinal detachment (RD) being the most common indication. In 2 patients (6.06%), the revision was due to intraocular bleeding (1 hyphema and 1 vitreous hemorrhage). During follow-up, 12 patients (36.36%) developed RD. Three cases (9.09%) occurred after silicone oil removal, while nine (27.27%) were associated with proliferative vitreoretinopathy (PVR). Retinal breaks were identified in only one case (3.03%), located inferiorly and related to PVR. Management involved pars plana vitrectomy and treatment of the underlying cause of RD, without the need for scleral procedures. Eight patients achieved successful RD repair after the first surgery, while four required a second intervention. Silicone oil was used as tamponade in 10 cases (30.30%), and long acting perfluoropropane gas in 2 cases (6.06%). Four patients (12.12%) experienced RD recurrence and required additional surgery; all had PVR during the initial intervention. By the end of follow-up, silicone oil had been removed in 23 patients (69.70%), with a mean retention time of 10.13 months.

Regarding procedure-related complications, macular edema was the most frequent, affecting 15 patients (45.45%). Treatment approaches included nepafenac and corticosteroids eye drops, intravitreal anti-VEGF injections, or intravitreal dexamethasone implants. Macular edema resolved completely in 4 patients (26.6%), showed partial improvement in 6 patients (40%), and did not respond to treatment in 5 patients (33.33%). Six patients (18.18%) presented significant epiretinal membrane, which was approached with pars plana vitrectomy during the silicone oil removal procedure or in a secondary procedure. Intraocular bleeding was observed in 12 patients (36.36%), with various types identified: scleral bed hemorrhage in 5 patients (15.15%), subretinal hemorrhage in 3 patients (9.09%), preretinal hemorrhage in 3 patients (9.09%), vitreous hemorrhage in 1 patient (3.03%), and hyphema in 2 patients (6.06%). Three patients (9.09%) presented submacular fibrosis. No patients presented phthisis bulbi. No patient required surgical intervention for glaucoma. Other complications included subretinal retained perfluorocarbon in 4 patients (12.12%), transient vertical diplopia in 1 patient (3.03%), and ocular hypertension in 2 patients (6.06%), one case secondary to neovascularization and the other secondary to silicone oil. In both patients, intraocular pressure elevation was successfully managed with topical medication containing two pharmacological agents.

Regarding brachytherapy, five patients (15.15%) suffered from radiation-related complications: 4 (12.12%) presented retinopathy and 1 (3.03%) neuropathy.

## 4. Discussion

In this study, we present our experience in the treatment of choroidal melanoma using a modified endoresection technique that intentionally leaves residual tumor margins up to 3 mm in height and includes adjuvant ^106^Ru brachytherapy. This approach aims to optimize functional outcomes—particularly visual acuity—without increasing rates of local recurrence, metastasis, or mortality. By avoiding complete resection down to the scleral bed, especially near critical visual structures like the fovea, this technique potentially reduces surgical trauma and intraoperative complications, thereby increasing eye-globe preservation and visual rehabilitation.

### 4.1. Risk of Tumor Dissemination

The concept of avoiding total tumor resection may raise concerns on an increased risk of dissemination due to the unconventional approach of a partial resection. However, multiple studies with long-term follow-up have not shown higher rates of metastasis or mortality compared to other treatment modalities [10,11,16,17,20,21]. Moreover, adjuvant radiotherapy is now considered standard in most endoresection protocols, targeting residual microscopic disease and reducing recurrence risk [2,6,10,16,17,18]. For instance, García-Arumí et al. reported no cases of local recurrence in patients who received ^106^Ru brachytherapy after endoresection, whereas 5 recurrences were noted in the group without adjuvant treatment [17]. Similarly, Biewald et al. observed fewer revisions and recurrences in cases where adjuvant radiotherapy was applied, though they also used preoperative stereotactic gamma knife therapy [16].

### 4.2. Prior Evidence on Residual Tumor Resection

Biewald et al. [16] reported a cohort of patients with choroidal melanoma, some of whom had ciliary body involvement—a location where achieving complete resection down to the scleral bed is technically more challenging. As a result, tumor remnants not exceeding 3 mm in height were intentionally left in certain cases. Although the exact number of such cases was not specified, the overall rates of local recurrence, enucleation, and melanoma-specific mortality were comparable to those reported in other series. These findings suggest that this more conservative surgical approach may be oncologically safe when used in conjunction with adjuvant therapy. Notably, a higher incidence of local recurrence was observed in tumors involving the ciliary body, consistent with previous evidence indicating that ciliary body melanomas typically have a worse prognosis than tumors confined to the choroid.

### 4.3. Tumor Control Metastasis and Survival

Long-term rates of local recurrence and enucleation after endoresection vary widely across different case series, ranging from 0% to 23% [6,10,18]. This variability may be attributed to differences in inclusion criteria, tumor size, genetic profile, and follow-up duration. In our study, despite intentionally leaving residual tumor margins, the rates of local recurrence and enucleation were 6.06% and 3.03%, respectively. By comparison, the COMS trial on medium-sized tumors reported cumulative local recurrence and enucleation rates of 8.3% and 8.7% at 42 months, both higher than the rates observed in our cohort [22].

Interestingly, greater tumor distance from both the fovea and optic disc was significantly associated with local recurrence in our series. A similar finding was reported by Papageorgiou et al., who noted an increased risk of recurrence with greater distance from the fovea [23]. However, other studies have suggested the opposite, associating tumors located near the fovea or in the posterior pole with higher recurrence rates [22,24]. Whether this discrepancy is related to intrinsic tumor characteristics or differences in surgical techniques remain uncertain.

Only one patient in our study died from metastatic disease during the follow-up period, resulting in a 5-year overall and disease-specific survival rate of 97%, according to Kaplan–Meier analysis. This outcome is comparable to the disease-specific survival reported in the long-term study by García-Arumí et al. [17]. Compared to the COMS medium-sized cohort, which reported 5-year metastasis-specific mortality rates of 10% and 6% at 42 months [4], our findings indicate a potentially lower metastatic risk in this selected population. Nevertheless, extended follow-up is essential to validate the long-term oncologic outcomes of this surgical approach.

### 4.4. Genetic Analysis and Prognosis

Cytogenetic profiling plays a critical role in the prognosis of uveal melanoma [25]. In our cohort, rates of monosomy 3 and 8q gain were at the lower end of the spectrum compared to other reports [26,27,28], suggesting a less aggressive tumor biology. Loss of chromosome 1p was the only genetic alteration significantly associated with both local recurrence and metastasis. This finding is consistent with the literature showing 1p loss as a common alteration in metastatic tumors and often coexisting with monosomy 3, together predicting worse disease-free survival [29,30,31,32,33].

### 4.5. Complications and Surgical Morbidity

Endoresection is inherently associated with both intraoperative and postoperative complications. However, the complication rates observed in our study were acceptable and, in some cases, lower than those reported in previous series. Intraocular bleeding occurred in 36.36% of patients, but no cases of phthisis bulbi, severe hypotony, or painful blind eye were documented. This contrasts with earlier reports such as Karkhaneh et al., where severe bleeding led to enucleation in 10% of cases despite the use of preventive measures such as endolaser photocoagulation and elevated intraocular pressure [34]. Similarly, García-Arumí et al. noted that all patients in their series experienced scleral bed bleeding, even with the application of diode laser beyond the tumor margins [17]. These findings support the hypothesis that leaving a small amount of residual tumor may help minimize surgical trauma and reduce the risk of severe intraoperative hemorrhage.

RD was the most frequent and clinically significant postoperative complication in our cohort, occurring in 36.36% of patients. This rate is slightly higher than those reported in most series [17,18,34,35,36]. For instance, Mazzini et al. observed an RD rate of 6.7% in a smaller cohort of 15 patients, using a 25-gauge vitrectomy platform and 3D visualization system, which may have contributed to greater surgical precision [2]. In contrast, Biewald et al., in a larger series of 200 patients, reported a RD rate of 29%, mostly attributed to PVR and comparable to the RD rate observed in our cohort [16]. In our series, PVR-related RD was also more prevalent than expected and often developed after silicone oil removal, requiring reoperation in 12.12% of patients.

Macular edema was another common complication in our series, affecting 45.45% of patients. This is consistent with previously reported rates ranging between 25% and 50% in other studies evaluating post-brachytherapy and post-resection outcomes [7,16]. Although frequently self-limiting or responsive to medical therapy, persistent macular edema can negatively affect final visual acuity, especially in eyes with otherwise stable anatomical outcomes. The high rate of edema in our cohort may reflect both surgical manipulation and radiation-related microvascular damage. Timely detection and treatment with corticosteroids or anti-VEGF agents may help preserve macular function in selected cases.

Collectively, these results imply that while retinal and macular complications remain significant concerns following endoresection, technical refinements and preventive strategies—such as early PVR management and edema monitoring—may improve anatomical and visual outcomes. The balance between tumor control and preservation of retinal integrity remains a central challenge in the evolution of this surgical approach.

### 4.6. Visual Acuity Outcomes

Functional preservation remains one of the principal objectives of this surgical approach. In our study, 60.61% of patients maintained a best-corrected visual acuity (BCVA) of 20/200 or better at final follow-up, and 27.3% achieved 20/40 or better. These outcomes are favorable when compared with the COMS trial on medium-sized tumors, where only 45% of patients retained BCVA ≥ 20/200 at three years [37]. This is particularly noteworthy considering that the COMS cohort included patients with smaller tumors, better baseline visual acuity, and fewer cases of preoperative retinal detachment.

Biewald et al., reported that 13.4% of patients had BCVA equal or better than 20/50 at the end of follow-up [16], and although visual acuity was not the primary outcome, the results suggested a significant loss of function in a substantial proportion of cases. In contrast, 27.3% of our patients had final BCVA equal or better than 20/40, despite many experiencing postoperative complications such as RD.

Mazzini et al. reported the most favorable anatomical and functional outcomes in a smaller cohort (*n* = 15), using advanced visualization systems and a 25-gauge vitrectomy platform [2]. Their results showed a lower incidence of RD and good preservation of central vision. Similarly to our results, García-Arumí et al. found that 48.8% of patients retained vision ≥20/200 [17].

Altogether, these findings suggest that the intentional preservation of tumor tissue near the fovea may protect delicate retinal structures and contribute to improved long-term visual outcomes. While this strategy may not be suitable for all cases, it offers a promising alternative for tumors located near critical visual areas.

### 4.7. Limitations and Future Directions

This study has limitations, including its retrospective design, single center study, small sample size, and limited follow-up time, which restricts conclusions about long-term safety, particularly the risk of metastasis. The lack of a control group limits direct comparison with standard endoresection techniques. Additionally, intraoperative estimation of residual tumor height is subjective and lacks objective validation.

Despite these limitations, the results are promising and consistent with other published reports. Future prospective, multicenter trials comparing this novel approach against conventional endoresection technique would provide more robust data to guide treatment decisions.

## 5. Conclusions

In conclusion, the modified endoresection technique with intentional preservation of residual tumor margins, combined with adjuvant ^106^Ru brachytherapy, appears to be a safe and effective treatment option for selected patients with choroidal melanoma. This approach may reduce surgical trauma and preserve visual function, without compromising local tumor control, metastasis risk, or overall survival. Despite a relatively high incidence of postoperative complications—particularly RD and macular edema—functional outcomes were encouraging, with the majority of patients maintaining useful vision. While these findings support the clinical value of a more conservative resection strategy, especially in tumors near critical visual structures, longer follow-up and prospective controlled studies are needed to confirm its long-term oncologic safety and functional benefits.

## Figures and Tables

**Table 1:** Clinical and demographic characteristics of enrolled patients (Quantitative variables).

	Mean (SD)	Median (IQR)	Min–Max
Age (years)	59.78 (12.58)	60 (20)	30–81
Visual acuity (Snellen Chart)	0.43 (0.38)	0.3 (0.7)	0.001–1
Visual Acuity (LogMAR)	0.95 (1.13)	0.52 (0.90)	0–3
Height (mm)	9.05 (1.98)	9 (1.8)	5.8–14
Tumor horizontal base (mm)	11.56 (2.47)	12 (4.5)	6.7–15.2
Tumor vertical base (mm)	11.19 (2.38)	11 (2.76)	7–16
Tumor maximum diameter base (mm)	12.05 (2.24)	12 (3.8)	7.6–16
Fovea distance (mm)	5.05 (2.7)	5 (2.3)	0–12
Optic nerve distance (mm)	4.38 (3.60)	5 (6.3)	0–14
Follow-up (months)	41.70 (17.98)	38 (27)	11–75

IQR = interquartile range, SD = standard deviation.

**Table 2:** Clinical and demographic characteristics of enrolled patients (Qualitative variables).

Baseline Characteristics	N (%)
Sex	
Men	20 (60.61)
Women	13 (39.39)
Eye	
Right	21 (63.64)
Left	12 (36.36)
Tumor size ^a^	
Medium	25 (75.76)
Large	8 (24.24)
Tumor size ^b^	
T2	11 (33.33)
T3	22 (66.67)
Exudative retinal detachment	
No	7 (21.21)
Yes	26 (78.79)
Tumor quadrant	
Superotemporal	2 (6.06)
Temporal	5 (15.15)
Inferotemporal	8 (24.24)
Inferior	4 (12.12)
Inferonasal	3 (9.09)
Nasal	4 (12.12)
Superonasal	6 (18.18)
Superior	1 (3.03)
Visual Acuity	
20/200 or better	25 (75.76)
Inferior to 20/200	8 (24.24)
Bruch membrane rupture	
No	2 (6.06)
Yes	31 (94.94)

^a^ According to COMS classification. ^b^ According to the 8th edition of AJCC staging.

**Table 3:** Treatment Outcomes and Follow-up.

Outcomes	N (%)
Metastatic disease	
No	31 (93.94)
Yes	2 (6.06)
Death	
No	32 (96.97)
Yes	1 (3.03)
Local recurrence	
No	31 (93.94)
Yes	2 (6.06)
Enucleation	
No	32 (96.97)
Yes	1 (3.03)
Complications	
Major revision surgery	12 (36.36)
Retinal detachment	12 (36.36)
Macular edema	15 (45.45)
Macular fibrosis	3 (9.09)
Bleeding	12 (36.36)
Epiretinal membrane	7 (21.21)
Post radiation retinopathy	4 (12.12)
Proliferative vitreoretinopathy	9 (27.27)
Final BCVA	
20/200 or better	20 (60.61)
Inferior of 20/200	13 (39.39)
Final BCVA (LogMAR)	
Median (IQR)	0.8 (1.5)
Follow-up (months)	
Mean (SD)	41.70 (17.98)

IQR = interquartile range, BCVA = best corrected visual acuity, SD = standard deviation.

## Data Availability

The raw data supporting the conclusions of this article will be made available by the authors on request.
